# Supplementary material for: Complete suspension culture of human induced pluripotent stem cells supplemented with suppressors of spontaneous differentiation
Source: eLife. 2024 Nov 12;12:RP89724. doi: 10.7554/eLife.89724 (PMC11556790; doi:10.7554/eLife.89724)
Supplement: Figure 1—figure supplement 1—source data 1. [file elife-89724-fig1-figsupp1-data1.zip › Figure1-Supplement1B_SourceData1.pdf]

PAX6-TEZ

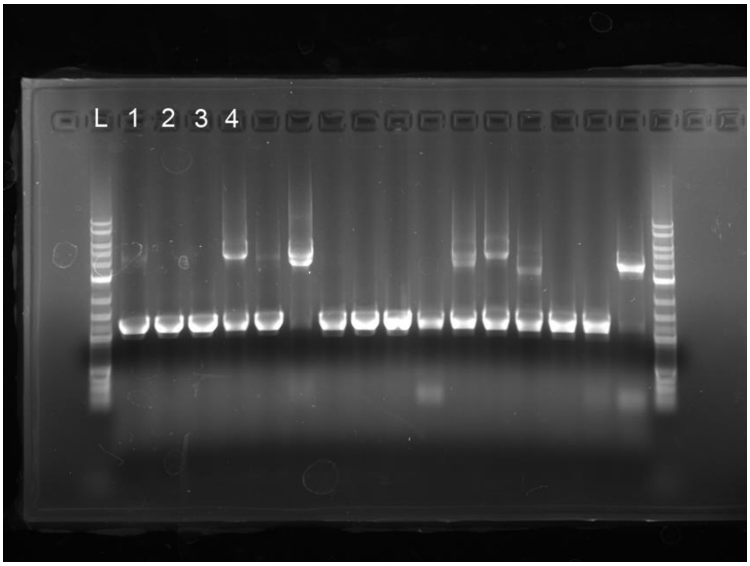

SOX17-TEZ

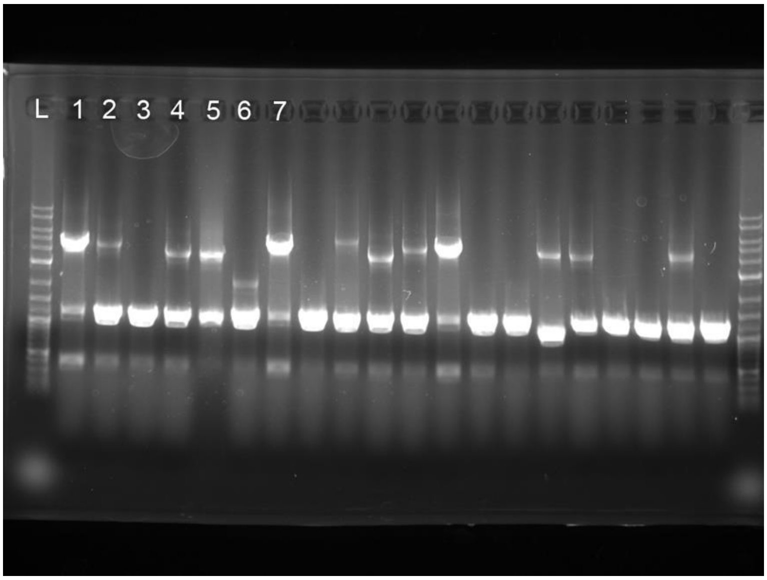

Figure 1—figure supplement 1, Source Data 1.
Original gel images corresponding to Figure 1—figure supplement 1, panel B.
