# Supplementary figures and images for: Complete suspension culture of human induced pluripotent stem cells supplemented with suppressors of spontaneous differentiation
Source: eLife. 2024 Nov 12;12:RP89724. doi: 10.7554/eLife.89724 (PMC11556790; doi:10.7554/eLife.89724)

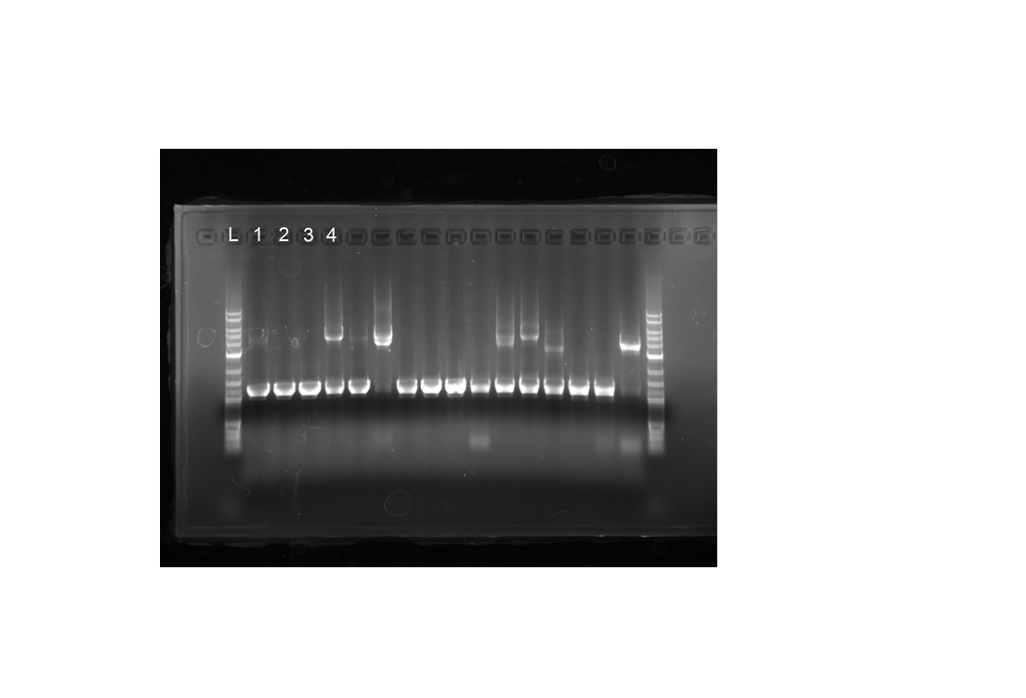

Supplement: Figure 1—figure supplement 1—source data 2. [file elife-89724-fig1-figsupp1-data2.zip › Figure1S1B_PAX6-TEZ.tiff]

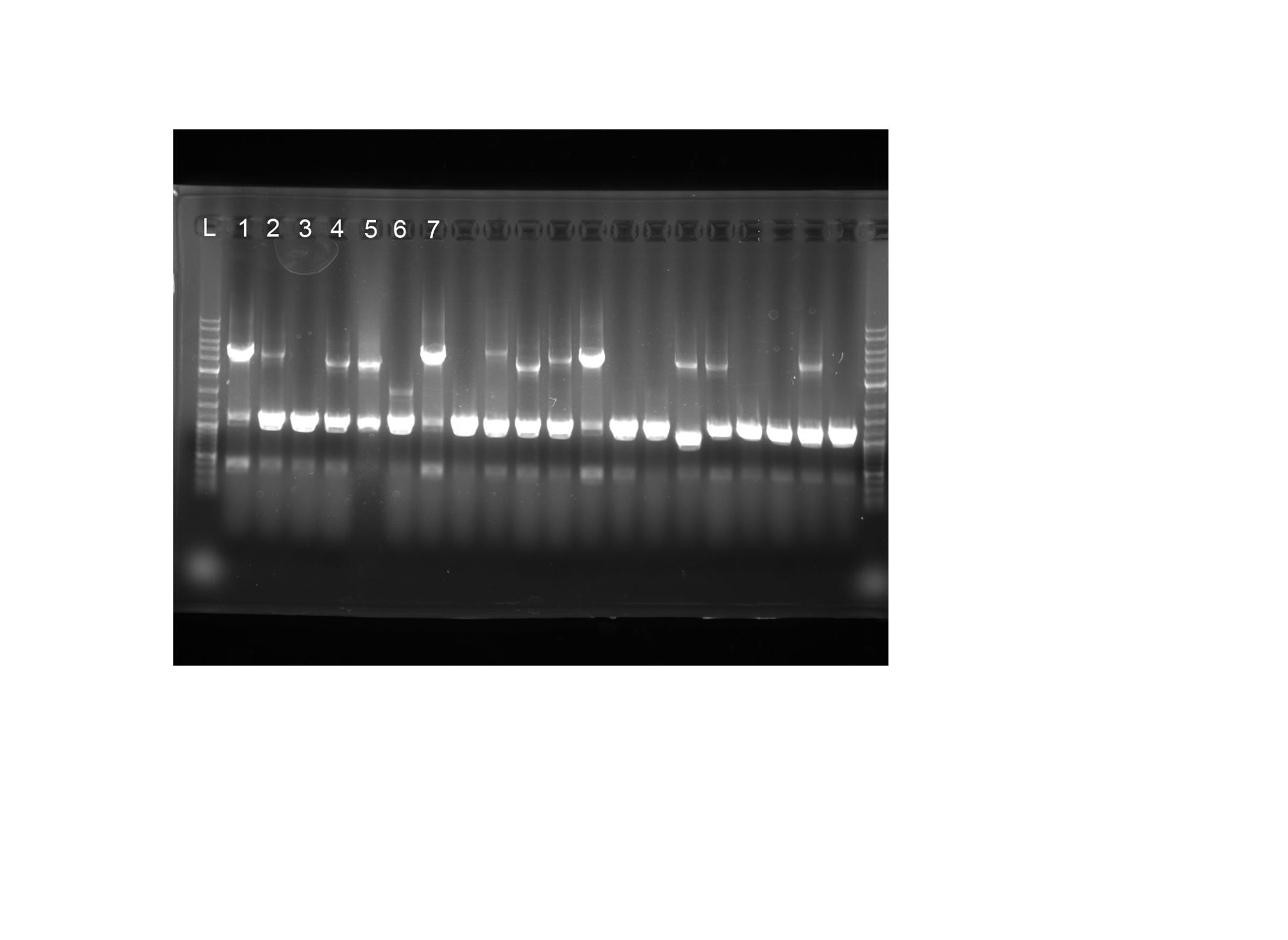

Supplement: Figure 1—figure supplement 1—source data 2. [file elife-89724-fig1-figsupp1-data2.zip › Figure1S1B_SOX17-TEZ.tiff]

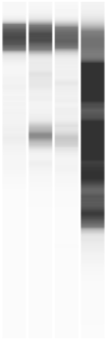

Supplement: Figure 2—figure supplement 1—source data 2. [file elife-89724-fig2-figsupp1-data2.zip › Figure2S1SsourceData1_Ex3_SOX17.tiff]

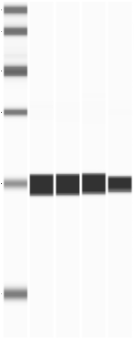

Supplement: Figure 2—figure supplement 1—source data 2. [file elife-89724-fig2-figsupp1-data2.zip › Figure2S1SsourceData1_Ex3_GAPDH.tiff]

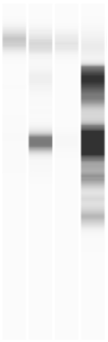

Supplement: Figure 2—figure supplement 1—source data 2. [file elife-89724-fig2-figsupp1-data2.zip › Figure2S1SsourceData1_Ex2_SOX17.tiff]

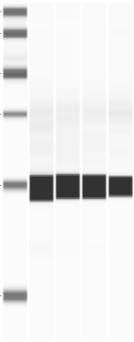

Supplement: Figure 2—figure supplement 1—source data 2. [file elife-89724-fig2-figsupp1-data2.zip › Figure2S1SsourceData1_Ex2_GAPDH.tiff]

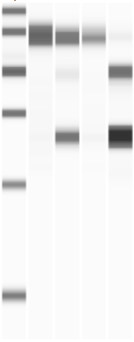

Supplement: Figure 2—figure supplement 1—source data 2. [file elife-89724-fig2-figsupp1-data2.zip › Figure2S1SsourceData1_Ex1_SOX17.tiff]

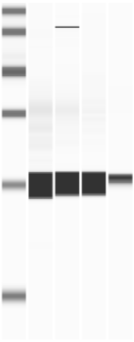

Supplement: Figure 2—figure supplement 1—source data 2. [file elife-89724-fig2-figsupp1-data2.zip › Figure2S1SsourceData1_Ex1_GAPDH.tiff]

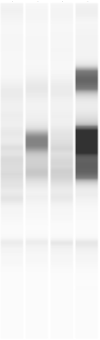

Supplement: Figure 2—figure supplement 1—source data 4. [file elife-89724-fig2-figsupp1-data4.zip › Figure2S1SsourceData3_Ex3_PAX6.tiff]

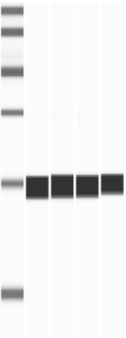

Supplement: Figure 2—figure supplement 1—source data 4. [file elife-89724-fig2-figsupp1-data4.zip › Figure2S1SsourceData3_Ex3_GAPDH.tiff]

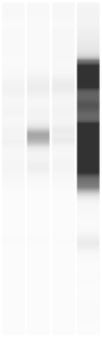

Supplement: Figure 2—figure supplement 1—source data 4. [file elife-89724-fig2-figsupp1-data4.zip › Figure2S1SsourceData3_Ex2_PAX6.tiff]

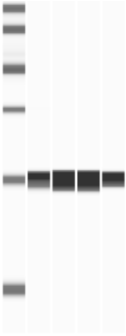

Supplement: Figure 2—figure supplement 1—source data 4. [file elife-89724-fig2-figsupp1-data4.zip › Figure2S1SsourceData3_Ex2_GAPDH.tiff]

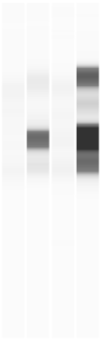

Supplement: Figure 2—figure supplement 1—source data 4. [file elife-89724-fig2-figsupp1-data4.zip › Figure2S1SsourceData3_Ex1_PAX6.tiff]

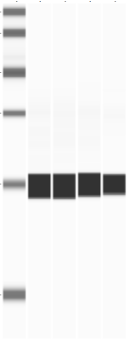

Supplement: Figure 2—figure supplement 1—source data 4. [file elife-89724-fig2-figsupp1-data4.zip › Figure2S1SsourceData3_Ex1_GAPDH.tiff]

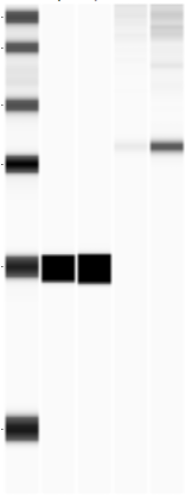

Supplement: Figure 3—figure supplement 1—source data 2. [file elife-89724-fig3-figsupp1-data2.zip › Figure3S1SourceData1_Ex1_GAPDH-pPKCb.tiff]

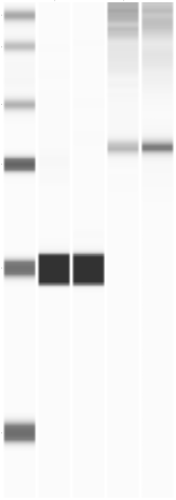

Supplement: Figure 3—figure supplement 1—source data 2. [file elife-89724-fig3-figsupp1-data2.zip › Figure3S1SourceData1_Ex2_GAPDH-pPKCb.tiff]

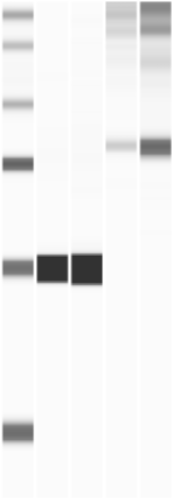

Supplement: Figure 3—figure supplement 1—source data 2. [file elife-89724-fig3-figsupp1-data2.zip › Figure3S1SourceData1_Ex3_GAPDH-pPKCb.tiff]
