# Supplementary material for: Complete suspension culture of human induced pluripotent stem cells supplemented with suppressors of spontaneous differentiation
Source: eLife. 2024 Nov 12;12:RP89724. doi: 10.7554/eLife.89724 (PMC11556790; doi:10.7554/eLife.89724)
Supplement: Figure 2—figure supplement 1—source data 1. [file elife-89724-fig2-figsupp1-data1.zip › Figure2-Supplement1A_SourceData1.pdf]

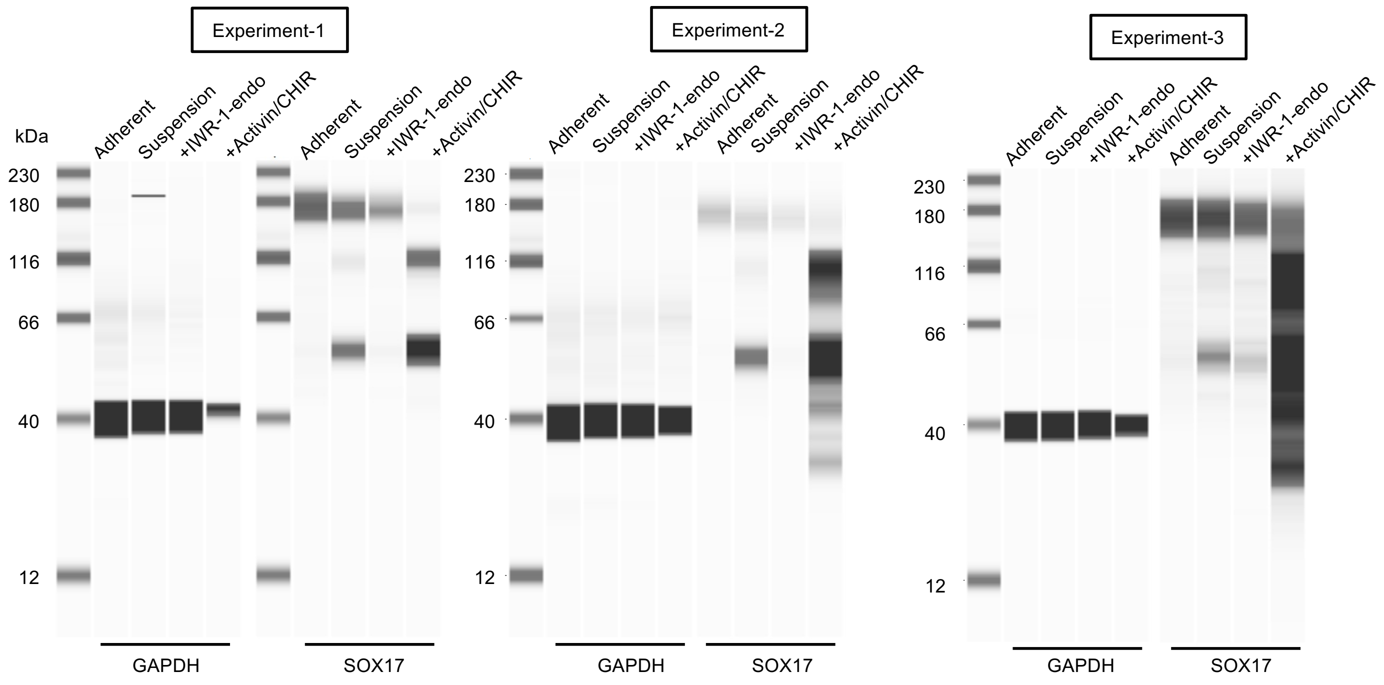

Figure 2—figure supplement 1, Source Data 1. Original automatic capillary western blots (simple western assays) corresponding to Figure 2—figure supplement 1, panel A.
