# Supplementary material for: Complete suspension culture of human induced pluripotent stem cells supplemented with suppressors of spontaneous differentiation
Source: eLife. 2024 Nov 12;12:RP89724. doi: 10.7554/eLife.89724 (PMC11556790; doi:10.7554/eLife.89724)
Supplement: Figure 2—figure supplement 1—source data 3. [file elife-89724-fig2-figsupp1-data3.zip › Figure2-Supplement1C_SourceData3.pdf]

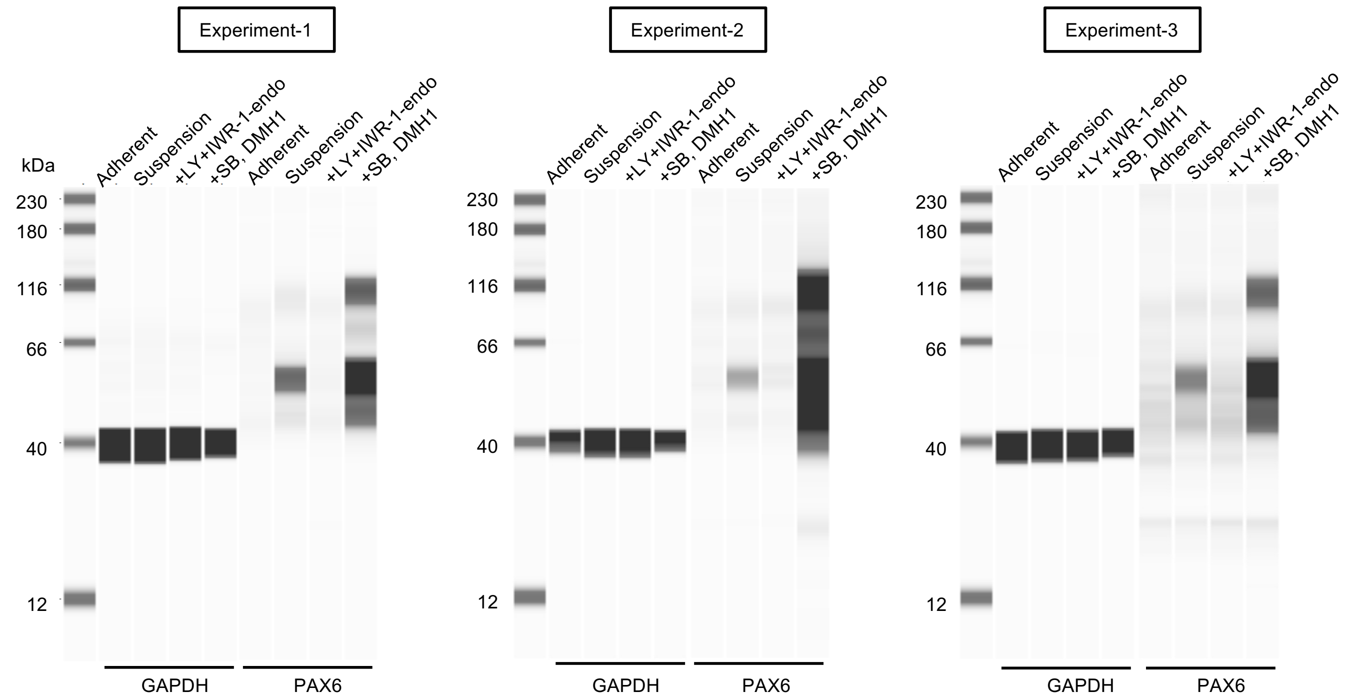

Figure 2—figure supplement 1, Source Data 3. Original automatic capillary western blots (simple western assays) corresponding to Figure 2—figure supplement 1, panel C.
