# Supplementary material for: Complete suspension culture of human induced pluripotent stem cells supplemented with suppressors of spontaneous differentiation
Source: eLife. 2024 Nov 12;12:RP89724. doi: 10.7554/eLife.89724 (PMC11556790; doi:10.7554/eLife.89724)
Supplement: Supplementary file 1. [file elife-89724-supp1.docx]

| **Name** | **Effect** | **Manufacturer** | **Identifier** | **Concentration** |
| --- | --- | --- | --- | --- |
| CHIR99021 | Wnt signal activator | Fujifilm Wako Pure Chemical | 034-23103 | 3 µM |
| IWP-2 | Wnt signaling inhibitor | Fujifilm Wako Pure Chemical | 034-24301 | 5 µM |
| IWR-1-endo | Wnt signaling inhibitor | Fujifilm Wako Pure Chemical | 037-25131 | 10 µM |
| Gö6976 | PKC (α, β) inhibitor | Fujifilm Wako Pure Chemical | 071-06551 | 5 µM |
| Gö6983 | PKC (α, β, γ, δ) inhibitor | Fujifilm Wako Pure Chemical | 078-06443 | 5 µM |
| GF109203X | PKC (α, β, γ) inhibitor | Tocris Bioscience | 0741 | 5 µM |
| Enzastaurin | PKC (α, β, γ, ε) inhibitor | Sigma-Aldrich | SML0762 | 5 µM |
| K-252a | PKC (µ) inhibitor | Sigma-Aldrich | K1639 | 5 µM |
| LY333531  (Ruboxistaurin) | PKC (β) inhibitor | Sigma-Aldrich | SML0693 | 1 µM |
| Myricitrin | PKC (α, ε) inhibitor | Sigma-Aldrich | 91255 | 5 µM |
| Sotrastaurin | PKC (α, β, δ, ε, η, θ) inhibitor | Cayman | 16726 | 5 µM |
| ZIP | PKC (ζ) inhibitor | Tocris Bioscience | 2549 | 5 µM |
| Ro-32-0432 | PKC (α, β, γ, ε) inhibitor | Tocris Bioscience | 1587 | 5 µM |
| FK506 | Calcineurin inhibitor | Fujifilm Wako Pure Chemical | 063-06071 | 100 ng/mL |
| Cyclosporine A (CsA) | Calcineurin inhibitor | Fujifilm Wako Pure Chemical | 031-24931 | 1 mg/mL |
| DAPT | γ-secretase inhibitor | Fujifilm Wako Pure Chemical | 043-33581 | 10 µM |
| BMP4 (Human, recombinant) | BMP-SMAD signaling activator | Fujifilm Wako Pure Chemical | 020-18851 | 40 ng/mL |
| LIF (Human, recombinant) | LIF-STAT signaling activator | Nacalai Tesque | NU0013-1 | 2000 units/mL |
| Valproic acid (VPA) | Epigenetic modifier | Fujifilm Wako Pure Chemical | 227-01071 | 0.5 mM |
| Ascorbic acid | Epigenetic modifier | Nacalai Tesque | 13048-42 | 50 ng/mL |
