# Supplementary material for: Complete suspension culture of human induced pluripotent stem cells supplemented with suppressors of spontaneous differentiation
Source: eLife. 2024 Nov 12;12:RP89724. doi: 10.7554/eLife.89724 (PMC11556790; doi:10.7554/eLife.89724)
Supplement: Supplementary file 2. [file elife-89724-supp2.docx]

| **Target gene** | **Taqman Assay ID or sequence** |
| --- | --- |
| *ACTB* | F: cctcatgaagatcctcaccga  R: ttgccaatggtgatgacctgg |
| *CDX2* | F: cacccacagccatagacctac  R: gtcagtccaggcaatgcttc |
| *CXCR4* | F: actgagaagcatgacggacaag  R: aggtagcggtccagactgatg |
| *GAPDH* | Hs02786624_g1 |
| *GATA6* | Hs00232018_m1 |
| *KLF2* | Hs07291763_gH |
| *KLF5* | Hs00156145_m1 |
| *NANOG* | Hs02387400_g1 |
| *OCT4 (POU5F1)* | Hs04260367_gH |
| *PAX3* | Hs00240950_m1 |
| *PAX6* | Hs01088114_m1 |
| *PDGFRa* | F: gctgagcctaatcctctgcc  R: actgctcacttccaagaccg |
| *PKCα (PRKCA)* | Hs00925200_m1 |
| *PKCβ (PRKCB)* | Hs00176998_m1 |
| *PKCγ (PRKCG)* | Hs00177010_m1 |
| *SOX1* | Hs01057642_s1 |
| *SOX2* | F: caccaatcccatccacactcac  R: gcaaagctcctaccgtaccac |
| *SOX17* | Hs00751752_s1 |
| *T* | Hs00610080_m1 |
| SeV detection | F: accaacaggcggtggtgcaa  R: tccaccccaacccctagcgt |
| *PAX6* genotyping | F: ggaattccagtacttcacgtgaaggcatct  R: tagaactgaagcggctctaacagccatttt |
| *SOX17* genotyping | F: agaaaggggtgcctttagaggacgggtgtt  R: aggaagtgtgtaacactgcttctggcctgc |
