# Supplementary material for: Complete suspension culture of human induced pluripotent stem cells supplemented with suppressors of spontaneous differentiation
Source: eLife. 2024 Nov 12;12:RP89724. doi: 10.7554/eLife.89724 (PMC11556790; doi:10.7554/eLife.89724)
Supplement: Supplementary file 3. [file elife-89724-supp3.docx]

| **Name** | **Host species** | **Dilutions or Concentrations** | **SOURCE** | **IDENTIFIER** |
| --- | --- | --- | --- | --- |
| Alpha-Fetoprotein (AFP) | Mouse | 1 µg/mL | R&D systems | Cat#MAB1368 |
| Alpha-SMA | Mouse | 1 µg/mL | R&D systems | Cat#MAB1420 |
| Cardiac Troponin T | Mouse | 10 µg/mL | Thermo Fisher Scientific | Cat#MA5-12960 |
| CORIN | Mouse | 10 µg/mL | Sigma-Aldrich | Cat#WH0010699M1 |
| GAPDH | Mouse | 1 µg/mL | R&D systems | Cat#MAB5718 |
| NANOG | Rabbit | 0.5 µg/mL | Reprocell | Cat#RCAB004P-F |
| OCT-3/4 | Mouse | 0.4 µg/mL | Santa Cruz | Cat#sc-5279 |
| Oct-4A (C30A3) Rabbit mAb (Alexa Fluor 488 Conjugate) | Rabbit | 1:30 | Cell Signaling Technology | Cat# 5177S |
| PAX6 | Rabbit | 1:400 (Immunocytochemistry) | MBL | Cat#PD022 |
| PAX6 | Sheep | 5 µg/mL (Wes) | R&D Systems | Cat#AF8150 |
| Anti-PKC beta1 + PKC beta2 (phospho T641) | Rabbit | 1:50 | Abcam | Cat#ab194749 |
| SOX17 | Goat | 0.4 µg/mL (Immunocytochemistry)  1 µg/mL (Wes) | R&D systems | Cat#AF1924 |
| FITC Mouse anti-SSEA-4 | Mouse | 1:5 | BD Bioscience | Cat#560126 |
| APC anti-human SSEA4 | Mouse | 1:20 | BioLegend | Cat#330418 |
| Alexa Fluor 488 anti-human TRA1-60-R | Mouse | 1:20 | BioLegend | Cat#330614 |
| Alexa Fluor 488 Mouse anti-Human TRA-1-60 Antigen | Mouse | 1:10 | BD Bioscience | Cat#560173 |
| TUJ1 | Mouse | 1 µg/mL | R&D systems | Cat#MAB1195 |
