# Supplementary material for: Complete suspension culture of human induced pluripotent stem cells supplemented with suppressors of spontaneous differentiation
Source: eLife. 2024 Nov 12;12:RP89724. doi: 10.7554/eLife.89724 (PMC11556790; doi:10.7554/eLife.89724)
Supplement: Supplementary file 4. [file elife-89724-supp4.docx]

| **Name** | **Host species** | **Dilutions** | **SOURCE** | **IDENTIFIER** |
| --- | --- | --- | --- | --- |
| Anti-Mouse IgG (H+L) Highly Cross-Adsorbed Secondary Antibody, Alexa Fluor Plus 488 | Donkey | 1:1000 | Thermo Fisher Scientific | Cat#A-21202 |
| DyLight 488 anti-rabbit IgG (minimal x-reactivity) | Donkey | 1:1000 | BioLegend | Cat#406404 |
| Anti-Mouse IgG (H+L) Highly Cross-Adsorbed Secondary Antibody, Alexa Fluor Plus 555 | Donkey | 1:1000 | Thermo Fisher Scientific | Cat#A-31570 |
| Anti-Rabbit IgG (H+L) Cross-Adsorbed Secondary Antibody, Alexa Fluor 555 | Goat | 1:1000 | Thermo Fisher Scientific | Cat#A-21428 |
| Anti-Mouse IgG (H+L) Cross-Adsorbed Secondary Antibody, Alexa Fluor 488 | Goat | 1:1000 | Thermo Fisher Scientific | Cat#A-11001 |
| Donkey Anti-Sheep IgG HRP Affinity Purified PAb | Sheep | 1:50 | R&D systems | Cat# HAF016 |
