# Supplementary material for: Complete suspension culture of human induced pluripotent stem cells supplemented with suppressors of spontaneous differentiation
Source: eLife. 2024 Nov 12;12:RP89724. doi: 10.7554/eLife.89724 (PMC11556790; doi:10.7554/eLife.89724)
Supplement: Supplementary file 5. [file elife-89724-supp5.docx]

| Name | Source | URL |
| --- | --- | --- |
| clusterProfiler (v3.16.1) | R package | https://bioconductor.org/packages/release/bioc/html/clusterProfiler.html |
| edgeR (v3.30.3) | R package | https://bioconductor.org/packages/release/bioc/html/edgeR.html |
| R (version4.1.0) | R package | https://www.r-project.org/ |
| CLC Genomics Workbench (v20) | QIAGEN | https://digitalinsights.qiagen.com/products-overview/discovery-insights-portfolio/analysis-and-visualization/qiagen-clc-genomics-workbench/ |
| SH800S cell sorter and flow cytometry software | Sony | https://www.sonybiotechnology.com/us/instruments/sh800s-cell-sorter/software/ |
| Compass software for Simple Western | Protein Simple | https://www.bio-techne.com/resources/instrument-software-download-center/compass-software-simple-western |
| BZ-X800 microscope analysis software | Keyence | https://www.keyence.com/landing/microscope/lp_fluorescence.jsp |
| QuantStudio 3 and 5 Real-Time PCR System Software | Thermo Fisher Scientific | https://www.thermofisher.com/jp/ja/home/global/forms/life-science/quantstudio-3-5-software.html |
| Chromosome Analysis Suite (ChAS) and Affymetrix GeneChip Command Console software programs | Thermo Fisher Scientific | https://www.thermofisher.com/jp/ja/home/life-science/microarray-analysis/microarray-analysis-instruments-software-services/microarray-analysis-software/chromosome-analysis-suite.html |
| CellSens microscope software | Evident (Olympus) | https://www.olympus-lifescience.com/en/support/downloads/ |
